# Knee Cartilage Lesion Management—Current Trends in Clinical Practice

**DOI:** 10.3390/jcm12206434

**Published:** 2023-10-10

**Authors:** Jaromir Jarecki, Marcin Krzysztof Waśko, Wojciech Widuchowski, Agnieszka Tomczyk-Warunek, Magdalena Wójciak, Ireneusz Sowa, Tomasz Blicharski

**Affiliations:** 1 Department of Orthopaedics and Rehabilitation, Medical University of Lublin, 20-059 Lublin, Poland; blicharski@vp.pl; 2 Department of Radiology and Imaging, The Medical Centre of Postgraduate Education, 01-813 Warsaw, Poland; marcin@wasko.md; 3 Department of Physiotherapy, The College of Physiotherapy, 50-038 Wrocław, Poland; sportmed@sportmed.com.pl; 4 Laboratory of Locomotor Systems Research, Department of Rehabilitation and Physiotherapy, Medical University of Lublin, 20-059 Lublin, Poland; a.tomczykwarunek@gmail.com; 5 Department of Analytical Chemistry, Medical University of Lublin, Chodzki 4a, 20-093 Lublin, Poland; magdalena.wojciak@umlub.pl (M.W.); ireneusz.sowa@umlub.pl (I.S.)

**Keywords:** cartilage, knee joint, mesenchymal stem cells, autologous chondrocyte implantation, microfracture, mosaicplasty, osteochondral autograft transfer, repair techniques, regenerative medicine

## Abstract

Many patients, particularly those aged above 40, experience knee joint pain, which hampers both sports activities and daily living. Treating isolated chondral and osteochondral defects in the knee poses a significant clinical challenge, particularly in younger patients who are not typically recommended partial or total knee arthroplasty as alternatives. Several surgical approaches have been developed to address focal cartilage defects. The treatment strategies are characterized as palliation (e.g., chondroplasty and debridement), repair (e.g., drilling and microfracture), or restoration (e.g., autologous chondrocyte implantation, osteochondral autograft, and osteochondral allograft). This review offers an overview of the commonly employed clinical methods for treating articular cartilage defects, with a specific focus on the clinical trials conducted in the last decade. Our study reveals that, currently, no single technology fully meets the essential requirements for effective cartilage healing while remaining easily applicable during surgical procedures. Nevertheless, numerous methods are available, and the choice of treatment should consider factors such as the location and size of the cartilage lesion, patient preferences, and whether it is chondral or osteochondral in nature. Promising directions for the future include tissue engineering, stem cell therapies, and the development of pre-formed scaffolds from hyaline cartilage, offering hope for improved outcomes.

## 1. Introduction

The knee joint, due to its location and complex function, is very susceptible to damage. Many patients, especially those over 40 years of age and sports patients after traumatic injuries, complain of painful symptoms in this joint, most commonly located in the medial compartment and the patellofemoral joint. These symptoms are caused by changes in the structure of the articular cartilage [1]. Articular cartilage is a highly differentiated and specialized connective tissue [2]. The extracellular matrix is mainly composed of water, collagen, and proteoglycans, as well as a small amount of non-collagenous proteins. Type II collagen is the most common form and represents about 90–95% of collagen in the extracellular matrix [3]. Articular cartilage is avascular and aneural, and lacks lymphatic vessels, resulting in a poor potential for healing. Damaged surfaces rubbing against each other accelerate the processes of the softening and cracking of the cartilage [4,5]. With progressive destruction of the cartilage, joint deformity occurs, leading to secondary damage to other structures, such as the menisci. Fragments of damaged menisci accelerate cartilage destruction and worsen pain [6]. The incidence of cartilage injuries is confirmed in over 60% of knee arthroscopies performed [7].

Articular cartilage lesions have poor repair capacity, leading to progressive joint damage, and cannot be predictably restored by conservative treatment, physical therapy, or injectable regimens.

Various methods are used to prevent the progression of cartilage lesions. One of them is orthotic treatment, which can correct existing deformities. Such treatment includes knee joint stabilizers, corrective insoles for shoes, special orthopedic shoes, and elbow crutches [8]. However, such treatment methods are not accepted for a long time, especially by young people. Another treatment method is the use of glycosaminoglycans. These substances are widely used in the treatment of cartilage injuries, but the results of therapy are highly variable [9]. Intra-articular injections of hyaluronic acid and platelet-rich plasma (PRP) can promote the healing of cartilage injuries and improve the lubricating properties of the joint [10]. However, these solutions have their limitations in cases of large cartilage defects and significant angular deformities of the knee joint [11]. In such situations, minimally invasive techniques based on the stimulation of bone marrow cells or the use of osteochondral grafts should be considered. These techniques are demanding, but failure to treat cartilage defects leads to the development of generalized cartilage lesions in the joint and ultimately ends in total joint replacement [12].

The aim of this review was to characterize the most commonly used minimally invasive surgical procedures to treat articular cartilage defects, based on evidence from clinical trials from the last decade.

Scopus, PubMed, Web of Science, and Google Scholar were utilized to identify papers related to the study’s objectives. The following keywords were used to search databases: “articular cartilage” OR “knee” OR “mesenchymal stem cells” OR “autologous chondrocyte implantation” OR “osteochondral defect” OR “microfracture” OR “mosaicplasty” OR “osteochondral autograft transfer” AND “repair techniques” OR “regenerative medicine”. The search period covered the period 2014 to 2023 and included searches in titles, abstracts, and keywords. All retrieved articles were reviewed based on inclusion criteria, which included full-text availability and English language. Excluded from the review were conference papers, reviews, abstract-only articles, books, and animal studies.

## 2. Surgical Strategies for the Treatment of Cartilage Defects

In clinical practice, various treatment options are available for addressing cartilage defects. However, surgical intervention should be specifically considered for symptomatic cartilage lesions graded as 3 or 4 according to the International Cartilage Repair Society (ICRS) classification [13]. Grade 3 lesions are characterized by defects exceeding 50% of the cartilage depth without extending into the subchondral bone, while grade 4 lesions involve both the cartilage and the underlying subchondral bone. These criteria are outlined in Table 1.

**Figure 1:**
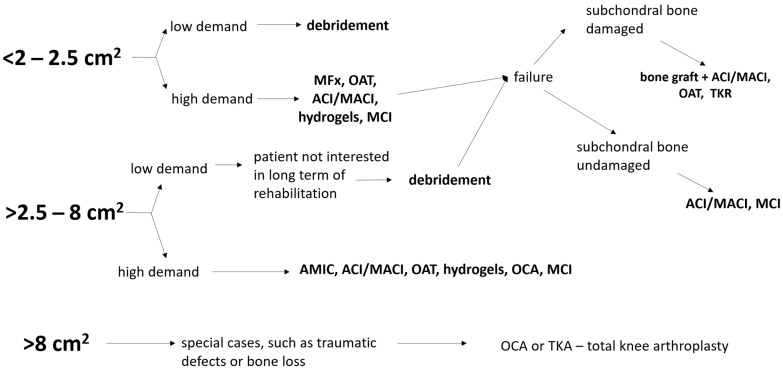
Approaches for the treatment of cartilage defects. MFx—microfracture, OAT—osteochondral autograft transfer, ACI—autologous chondrocyte implantation, TKA—total knee arthroplasty, OCA—osteochondral allografts, MCI—minced cartilage implantation, MACI—matrix-assisted chondrocyte implantation, AMIC—autologous matrix-induced chondrogenesis.

Treatment options for chondral lesions involve either marrow-stimulating techniques or cell-regenerating approaches. Osteochondral lesions, on the other hand, can be addressed through bone graft and chondrocyte implantation, mosaicplasty, or allografts. It states that the choice of surgical procedure depends on factors such as the location and size of the cartilage lesion, patient demands, and whether it is chondral or osteochondral in nature. The size of the lesion is typically determined post debridement, and it is measured by area (cm^2^) (width × length of lesions). The location and size of the defect are preoperatively estimated using MRI and definitively assessed during arthroscopy. Traditionally, defects larger than 8 cm^2^ are not considered suitable for regenerative surgery. The scheme of approach for the treatment of cartilage defects is shown in Figure 1.

The knee joint consists of two articulations: the patellofemoral and tibiofemoral joints. They differ from each other in terms of shape, biomechanics, and the pressure generated during movement. The treatment of cartilage defects in these joints is similar, but the postoperative management varies. Rehabilitation after surgery should consider the anatomical differences of both joints, with particular attention to the location and extent of the injury, as well as the surgical technique used.

The cartilage lesions lead to focal degeneration, however, a disturbed joint homeostasis after, for example, trauma, may induce a generalized loss of cartilage, leading to end-stage osteoarthritis, encompassing the entire joint. Interestingly, isolated articular cartilage defects seem to be associated with general knee osteoarthritis severity [14]. In recent years, regenerative medicine has been applied to patients who have clinically evident but not end-stage osteoarthritis. It seems that new regenerative technologies can work better for focal lesions when the overall joint damage is minimal [15].

### 2.1. Microfracture, Drilling, and Abrasion

Techniques stimulating bone marrow cells are widely used in the arthroscopic treatment of cartilage and osteochondral defects. Among them, microfracture, drilling, or abrasion of the subchondral layer should be mentioned. The aim of these therapies is to induce the extravasation of bone marrow rich in stromal cells and the secondary formation of a fibrin clot, which will be the basis for creating hyaline cartilage in the defect site [16]. However, bone marrow cells may differentiate into fibrochondrocytes, resulting in the formation of fibrous cartilage, sometimes with elements of fibrocartilage. Fibrous cartilage is mainly composed of type I collagen and has weaker biochemical and biomechanical properties than hyaline cartilage [17].

Microfracture (MFx) used in the treatment of cartilage defects is often considered the gold standard of therapy. The first results and description of the microfracture technique were published by Steadman in 1994 [18]. The idea of the method is to support the natural process of healing the cartilage defect by releasing bone marrow stromal cells. Initially, microfracture was performed in patients with full-thickness cartilage defects resulting from knee joint injuries. The indication for the use of this treatment method was also unstable damage to the cartilage covering the subchondral layer, as well as cartilage lesions in the knee joint with preserved anatomical mechanical axis [19]. The microfracture technique is the simplest and cheapest method of treating osteochondral defects and is used as a first-line procedure in the treatment of such injuries [20]. It involves creating 4 mm-deep holes throughout the surface of the cartilage defect, which are 3–4 mm apart from each other [21]. Special tools are used to perform the microfracture, which can be used in both open and arthroscopic techniques. The defect should be cleaned of any remaining damaged cartilage to expose the subchondral layer and obtain a stable environment for the defect through healthy, undamaged cartilage. Such preparation of the defect provides stability for the developing clot, which contains bone marrow cells [22].

Subchondral drilling or abrasion is an alternative method to microfracture and involves drilling the damaged surface with a special drill or thin Kirschner wire [23]. Subchondral abrasion requires the use of a drill bit, which allows for the removal of the damaged cartilage tissue and partially removes the subchondral bone layer. This technique is less commonly used than microfracture because it can cause thermal damage to osteocytes, as well as excessive damage to the subchondral layer, resulting in necrosis, hypertrophy, or the development of intraosseous cysts [21]. However, animal studies have shown that drilling does not destroy more osteocytes than microfracture [24].

Nanofracture is a variation of microfracture that differs from the primary technique in terms of the smaller size of the hole, of up to 1 mm, and deeper penetration, of up to 9 mm. The advantage of this method is the possibility of a denser distribution of microfracture and less damage to the subchondral layer at the site of the defect. Additionally, osteocytes are not thermally damaged during the procedure. Animal studies confirm less destruction of trabecular bone in the case of nanofracture, as well as less crushing and fragmentation of the subchondral layer. Moreover, better healing and anatomical reconstruction of trabecular bone and less frequent formation of subchondral cysts are observed [25].

On the other hand, deeper subchondral penetration results in more effective filling of the defect by the extravasated blood and proportionally more frequent development of fibrocartilage at the site of injury [26].

Over the years, various microfracture techniques have been thoroughly investigated. Many studies have confirmed an improvement in clinical outcomes in early postoperative observations [27]. However, the relatively common development of fibrous cartilage and worsening of results in long-term observations should encourage considerations of alternative surgical techniques, including autologous chondrocyte implantation and osteochondral cylinder transfers. [28].

An alternative to these techniques includes the augmentation of microfractures with biological agents. For example, microfracture augmented with particulated costal allocartilage resulted in superior cartilage repair quality compared with microfracture alone at short-term follow-up [29]. In general, biological augmentation resulted in significant improvements in patient-reported outcome measures [30,31,32]. However, in all the trials, this improvement did not reach the minimally clinically important difference, meaning that it was not perceivable by the patients. Moreover, the overall low evidence and the paucity of high-level studies indicate further research is needed to confirm the potential of PRP augmentation to microfracture for the treatment of cartilage lesions [33].

In summary, many studies have confirmed that microfracture brings better clinical results in the treatment of smaller defects involving an area of 2–4 cm^2^ and in younger patients [34].

### 2.2. Autologous Matrix-Induced Chondrogenesis

A fibrous clot formed as a result of microfracture does not have proper mechanical stability and is not able to withstand the shearing forces that occur during joint movements [35]. To prevent damage to the clot formed after microfracture, the autologous matrix-induced chondrogenesis (AMIC) technique is used. This technique combines microfracture with a collagen patch covering the defect, which provides mechanical stability to the clot and allows stromal cells to differentiate into chondrocytes. To seal the defect, the collagen patch is stabilized with cartilage using tissue adhesives or sutures. Randomized clinical trials comparing microfracture with AMIC showed that the tissue produced was quantitatively and qualitatively better in 5-year observations in the AMIC group. However, no clear differences in clinical outcomes were observed [36]. The best results with this method are obtained when it is used in defects of up to 12 cm^2^.

### 2.3. Techniques Based on Chodrocyte Implantation

In 1970, Bentley and Greer conducted animal studies in which they implanted chondrocytes into cartilage defects with the aim of restoring hyaline cartilage [37]. This method, known as autologous chondrocyte implantation (ACI), was developed in the 1990s by Brittberg et al. and is divided into two stages [38]. The first stage involves harvesting articular cartilage from an unburdened area of the joint. Chondrocytes are released from the cartilage under enzymatic digestion in laboratory conditions. Then, they are multiplied for a period of 4 to 6 weeks. The second stage involves implanting the multiplied chondrocytes into the cartilage defect. In the original method, chondrocytes were delivered under a periosteal patch, which was sutured to the surrounding cartilage and additionally reinforced with fibrin glue. A characteristic feature of this method was periosteal overgrowth, which resulted in painful popping observed in about 25% of patients [39]. Currently, periosteal patches have been replaced by various types of collagen patches.

MACI—Matrix-Assisted Chondrocyte Implantation is a second-generation technique that uses scaffolds made from a mixture of collagen types I and III. Chondrocytes are grown on these scaffolds, which are then implanted at the site of the defect and stabilized with tissue glue [40]. This technique shortens the surgery time and avoids the complications that were associated with the use of periosteal membrane patches. The next stage in treatment will involve using chondrocyte cultures in the form of small spheres, called chondrospheres [41].

In both ACI and MACI methods, the rebuilt cartilage has a hyaline or hyaline-like structure in histological studies. In 5-year observations, the graft survival rate oscillates around 78%, while in 10-year observations, it is 51%. The improvement in functional results is comparable to mosaicplasty [42,43]. This is confirmed, among others, by studies by Minas and colleagues, who presented a graft survival rate of 71% in 10-year observations and an improvement in function in 75% of cases in patients with defects of an average size of 8.4 cm^2^. ACI/MACI has been approved and recommended by the National Institute for Health and Care Excellence (NICE) in the UK as a first-line treatment for cartilage defects. ACI/MACI is recommended for large cartilage defects with a surface area of up to 22 cm^2^ [44]. Despite its many advantages, this method also has some drawbacks, including the need for two surgical procedures, a long rehabilitation time after surgery, and high surgical costs.

The classic ACI technique has been modified in recent years, and three-dimensional matrix-based procedures (e.g., spheroids) have been developed. Spheroids are a type of three-dimensional cell aggregate, self-assembling under conditions that prevent attachment to a flat surface [45]. Once formed into a self-adhesive matrix, the spheroids (marketed under the name Spherox by CO.DON AG) are implanted into the defect, and the chondrocytes migrate and synthesize extracellular matrix components, thus filling the lesion [46]. There are promising mid- to long-term results of this technique [47,48]; the histological and immunohistological outcomes are excellent at 6 to 16 months after the surgery, showing the regeneration of hyaline articular cartilage [49]. However, there still remain questions regarding the optimal cultivation time [50] and dosage [51], and operating surgeons should be aware of the frequent adverse reactions, including joint effusion, arthralgia, and joint swelling [52]. 

Another approach to cartilage regeneration utilizes rapidly isolated recycled autologous chondrons (chondrocytes with pericellular matrix [53,54]) with allogeneic MSCs in a one-stage surgery. So far, animal test results have been very promising [55]. There was a single, proof-of-concept, first-in-human study by Saris et al. [56]. In the study, no signs of a foreign body response or serious adverse reactions were recorded after 5 years in 35 of the study group patients. The majority of patients showed statistically significant and clinically relevant improvements in the KOOS and all of its subscales from baseline to 60 months. The authors concluded that these data support MSC-augmented chondron transplantation (coined IMPACT) as a safe one-stage surgical intervention. However, one should await the results of the larger-scale human trial, for which a study protocol was published in 2020 [57].

### 2.4. Hydrogels Treatment

Another method used in the treatment of cartilage defects is hydrogels. It is one of the more modern techniques that can be used in both open and arthroscopic methods. Hydrogels have different characteristics regarding reabsorption time. When this time is fast, the migration of stromal cells may not be sufficient to restore the correct structure of the cartilage defect, while in the case of too slow bioabsorption, chondrocyte overgrowth may cause irregularities on the surface of the joint cartilage. Due to these limitations, the use of gels is currently combined with the microfracture technique, which improves the quality of the resulting cartilage tissue. Hydrogels are characterized by good lubricating properties as well as biomechanical characteristics that allow stromal cells to mature. The efficiency of gels depends on their mechanical strength and modular elasticity. The first gels did not have sufficient compressive strength and load-carrying capacity, which appeared during the normal functioning of the knee joint and did not fulfill their stabilizing role for mesenchymal cells. Currently, improved gel preparations have very good mechanical properties. Shive et al. evaluated the early and 5-year results of gel use compared to microfracture. They obtained significantly better MRI image results in the group using the gel for filling and healing the cartilage defect, while in 5-year observations, the WOMAC scale results were comparable in terms of pain, stiffness, and function [36].

### 2.5. Osteochondral Autograft Transfer Mosaicplasty

Osteochondral autograft transfer (OAT) mosaicplasty is a surgical technique in which cartilage-bone cylinders are taken from non-weight-bearing zones of the joint and are simultaneously transplanted and implanted in the area of the cartilage defect after its preparation. The bone portion of the autograft provides an excellent foundation and stability for the cartilaginous portion above it. In the procedure, one or several cylinders can be used to fully fill the cartilage defect. However, this technique is more time-consuming than microfracture and requires the use of special tools. The bone fragment of the graft usually fully fuses with the surrounding bone, while the cartilage fragment does not always undergo biointegration with the surrounding cartilage. Gaps appear between the cartilage-bone cylinders, which are filled with fibrous tissue that transforms into fibrocartilaginous tissue. This also does not achieve homogenous uniformity in the filled defect. The disadvantage of mosaicplasty is the potential mismatch of the size and shape of the graft compared to the filled defect [58]. Despite its drawbacks, this technique has significantly better long-term outcomes than microfracture. Opinion papers that used mosaicplasty showed very good results, with a survival rate of 72% in observations over 10 years [59]. However, the older age of patients, previous surgeries, and larger cartilage defects ultimately affect worse long-term outcomes [60]. These findings are confirmed by Bentley’s studies, which found mosaicplasty to be less effective than ACI in large cartilage defects. However, mosaicplasty has significantly better outcomes in smaller defects compared to ACI [61].

### 2.6. Fresh Osteochondral Allografts

Fresh osteochondral allografts (OCA) are an alternative to OATS. They can be used in cases where the primary treatment of cartilage defects with other methods has failed [62]. The advantage of this method is that it avoids harvesting material from non-weight-bearing areas and allows for coverage of large surface areas that would not be possible with autologous grafts under normal conditions. The value of this method has been confirmed in numerous studies for the treatment of both local and extensive injuries in demanding patients [63,64]. The survival rate of grafts in this method has been reported to be up to 82% in 10-year follow-up observations and 74% in 15-year follow-up observations [65]. The correct storage of fresh osteochondral allografts at physiological body temperature is crucial for the ultimate treatment outcome, as it significantly affects the survival of chondrocytes [20]. The main problem with this technique is the possible rejection reaction of the graft by the recipient. Additionally, thin grafts of less than 1 cm or smaller may not heal, and the cartilage may not consolidate with the graft. Another problem with the use of fresh osteochondral allografts may be their availability [66]. 

### 2.7. Stromal Cells

Mesenchymal stromal cells (MSCs) are unspecialized, primary cells with a high potential for proliferation and transformation into specialized cells. Depending on their origin, stromal cells can be divided into embryonic stromal cells and adult stromal cells. Until now, stromal cells have been administered through intra-articular injections to slow down the degenerative process of the joint. Currently, stromal cells are being explored to be combined with other types of therapies for the potential development of hyaline cartilage regeneration. The administration of stromal cells is used either during the filling of a cartilage defect at the site of the lesion or delivered into the joint as a means to enhance tissue healing. The use of bone marrow stromal cells to repair cartilage defects can be performed as a one-step procedure, which makes it significantly cheaper than ACI/MACI techniques [67]. The injection of stromal cells into the joint for the treatment of cartilage defects has not been fully confirmed, but reports appear in the literature indicating the beneficial effects of such therapy in cartilage lesions and aseptic necrosis of the bone. 

A review of the literature confirms that intra-articular injections of stromal cells derived from adipose tissue improve clinical scores, magnetic resonance imaging, arthroscopic evaluation, and histological evaluation of cartilage material obtained after stem cell treatment [68]. Additionally, better results have been obtained in studies comparing the effectiveness of stromal cells to hyaluronic acid [69]. Sekiya et al. combined stromal cells taken from the synovial membrane with patient serum and administered them under arthroscopic control to the sites of cartilage defects, observing improvements in clinical outcomes and MRI images [70]. So far, few studies have been published on the combination of stromal cells with scaffolds as a simultaneous procedure. Most published studies so far have been patient series. Buda et al. transplanted stromal cells in combination with a hyaluronic acid membrane in cartilage defects of the medial and lateral femoral condyles in 20 patients. Researchers observed a significant improvement in clinical symptoms in 2-year observations [71].

Nejadnik et al. compared a group of patients who underwent ACI with a group of patients treated with stromal cells. Overall, the treatment outcomes in both groups were comparable and indicated an improvement in the quality of life of patients, as well as significantly improving the physical activity of the subjects [72]. However, the use of stromal cells in combination with collagen membranes still requires randomized, multicenter clinical trials to be conducted.

### 2.8. Autologous Cartilage Repair Technique

Minced cartilage implantation (MCI) is another promising cartilage reconstruction technique. It consists of transplanting autologous cartilage fragments in a single-step procedure. First, cartilage from the lesion, less weight-bearing areas, or both are harvested, optimally preserving the uniform fragment size without crushing the fragments. Simultaneously, autologous thrombin and platelet-rich plasma (PRP) solutions are prepared. After the defect is cleaned and dried, cartilage fragments and PRP are introduced and stabilized in situ with autologous fibrin glue, membrane, or staples [73]. Since adult human cartilage cells are postmitotic, they cannot fill the cartilage defects [74]. However, the fragmentation of healthy cartilage is meant to activate mitogenic activity, and following implantation, an outgrowth of autologous chondrocytes is initiated. The outgrowth of chondrocytes from minced pieces results in proliferation, which is hoped to promote the extracellular matrix (ECM) production of naive articular cartilage tissue [75].

In animal models, minced cartilage has shown results superior to membranes and microfracture, similar to ACI [76]. In addition, chondrocyte outgrowth from chondral fragments has been proven feasible in vivo, further suggesting the viability of this technique [77].

There is only limited clinical evidence on autologous minced cartilage procedures in humans [78,79]. While the published studies have shown satisfying clinical outcomes and safety, with failure and revision rates comparable to other available cartilage repair techniques, more comparative trials are necessary to allow comparison with alternative cartilage repair techniques. Autologous minced cartilage repair does not require manipulating the specimen in the laboratory or using allografts. It is, therefore, economically attractive and should not require significant regulations. In summary, minced cartilage has a strong biologic potential since autologous, activated, non-dedifferentiated chondrocytes are utilized. Thus, one avoids the problems of the dedifferentiation of chondrocytes, which produces weak type I collagen, not primarily associated with healthy cartilage, while maintaining adequate cellularity required for matrix generation. Preliminary data show that it can be used for small and large cartilage and osteochondral lesions. However, comparative study and long-term outcomes data are required to the determine minced cartilage construct’s effectiveness and durability.

### 2.9. Scaffold-Based Therapies

Scaffold-based therapies emerged as another potential solution for chondral and osteochondral lesions. Primarily, scaffolds were implanted with autologous chondrocytes, replacing the periosteal patch to contain the cell culture, and later as matrix-assisted ACI, serving as a culturing medium [80,81,82]. However, cell-based therapies involved regulatory restrictions, higher costs, and required an additional surgical procedure for cell harvest and time for cell expansion; cell-free scaffolds were thus introduced. The cell-free approach was meant to allow native cells to populate the scaffold over time [83]. Along with increasing the body of evidence, two approaches have recently dominated clinical practice—a trend for a single-step procedure with various cell sources and the use of biomaterials as a stand-alone, cell-free therapy [81,84]. The latter approach also marks clinicians’ increasing understanding that scaffolds could not only serve as a matrix for transplanted cells, but can also display intrinsic properties supporting tissue regeneration, promoting chondral and osteochondral regrowth [84]. 

A variety of scaffolds have been introduced to clinical practice and there are human studies showing good mid- to long-term results, regardless of the scaffold form (fibers, gels, meshes) and cell content (cell-free or including autologous chondrocytes) [85,86]. Importantly, both clinical outcomes and the return to sports rates for scaffold-based techniques are similar to other repair techniques; for example, ‘kissing lesions’ are connected with inferior results for this technique too [82,87,88]. This finding, present in many studies on cartilage repair, signifies the influence of both biomechanics as well as the overall joint status (arthritic vs. non-arthritic) on the outcomes of different cartilage restoration procedures.

## 3. Novel Techniques

Novel techniques are being introduced into clinical trials to improve the clinical outcomes of cartilage lesion treatment. Articular chondrocyte-based autologous chondrocyte implantations have been shown to restore articular cartilage defects. However, there is a significant donor-site morbidity associated with this technique, which increases the likelihood of developing osteoarthritis [89,90], and the available volume of chondral tissue is limited. Therefore, alternative cell sources for cartilage repair have been introduced, including nasal, auricular, and costal cartilage [91,92]. The trials performed in humans suggest that these innovative techniques can become an established therapeutic option, while overcoming the limitations of classical ACI procedures. Short- to mid-term results have been promising, with good clinical outcomes and confirmed structural regeneration [92,93]. The evolution of these techniques could also involve 3D bioprinting in the future [94].

In the last decade, numerous scientific reports have been dedicated to describing the clinical observation of the effects of therapy using different approaches (Table 2). Furthermore, some papers have compared the effectiveness of various treatments for knee cartilage defects. Some examples of the observed effects of therapy are presented in Table 3.

## 4. Conclusions

In summary, this article outlines the current treatment options for articular cartilage injuries, as well as their indications, advantages, and limitations. Furthermore, it explores future prospects in cartilage regeneration techniques. The dynamic advancements in science and biotechnology provide the groundwork for creating superior materials for knee joint cartilage defect treatments. Tissue engineering takes the lead, aiming to develop tissues that mimic the biological, structural, and functional characteristics of hyaline cartilage, with better integration into the surrounding tissue. Consequently, this could enhance the longevity of implants in the mechanically demanding knee joint environment.

Stem cell therapies hold great promise as readily available and biologically compatible materials. Promising advances in gene therapies and the development of pre-formed scaffolds from hyaline cartilage offer hope for the future. Overall, there is currently no technology that fully meets the essential requirements for effective cartilage healing, which include proper extracellular matrix organization and bioactivity, and can be easily applied by surgeons during surgical procedures. However, the latest techniques, although not yet widely available in clinical practice, provide hope for the future. 

## Figures and Tables

**Table 1:** Classification of knee cartilage defects according to ICRS (International Cartilage Repair Society Classification).

Score		Clinical Description
0		Normal cartilage
1	a	Soft indentation
	b	Superficial fissures and cracks
2		Lesions extending down to less than 50% of the cartilage depth
3	a	Defects extending down to more than 50% of the cartilage layer
	b	Defects extending down to the calcified layer
	c	Defects extending down to but not through the defects extending down to subchondral bone layer
	d	Delamination, including bulging of the cartilage around the lesion
4	a	Penetration of the subchondral bone but not across the entire diameter of the defect
	b	Penetration of the subchondral bone across the full diameter of the defect

**Table 2:** Examples of the application of different surgical strategies in the treatment of knee cartilage defects.

Treatment	Patients (m/f)	Lesion Size (cm^2^)	Follow-Up	Clinical Outcome	Ref.
Microfracture (MFx)	43/18	2–6	15.1 years (10–20 years)	IKDC and LS increased: 46.7 ± 2.9 to 71.5 ± 4.0, 45.4 ± 3.5 to 77.2 ± 3.5, respectively; Tegner average scores decreased at final follow-up from 7 to 4. Patients with smaller lesions (≤4 cm^2^) and younger (≤30 years) showed better results in KOOS, VAS, and Marx scores.	[95]
Microfracture (MFx)	49/32	2.295 (0.25–20.00)	2.6 years (2–5 years)	Pain, swelling, limping, walking, stairs, sport level, and activities of daily living improved over preoperative status (*p* < 0.003). LS improved from 53.8 to 83.1, and the mean Tegner Activity Scale score improved from 2.9 to 4.5.	[96]
Microfracture (MFx)	37/33	2.0–2.39	36 months	MRI after surgery revealed best defect filling in lesions on the femoral condyles with significant difference in other areas (*p* < 0.02). The Pearson coefficient of correlation between the defect filling and ICRS score was 0.84 and significant at the 0.01 level.	[97]
AMIC^®^ glued AMIC^®^ sutured Microfracture (MFx)	15/2 12/5 10/3	3.9 ± 1.1 3.8 ± 2.1 2.9 ± 0.8	5 years	MRI defect filling was more complete in the AMIC^®^ groups. The Cincinnati score increased from 38± 19 for the MFx, 48 ± 15 for the glued AMIC^®^, and 45 ± 19 for the sutured AMIC^®^ to 72 ± 18 (*p* < 0.001), 67 ± 26 (*p* = 0.028), and 82 ± 15, respectively, at one year post operation. After five years, the Cincinnati score was at least stable or even improving in both AMIC^®^, whereas a significant decrease was observed in the MFx ICRS for pain decrease in all groups from 57 ± 22, 46 ± 20, and 54 ± 19 for MFx, the glued AMIC^®^, and the sutured AMIC^®^, respectively, to 15 ± 17 for MFx, 15 ± 13 for glued AMIC^®^, and 16 ± 15 for the sutured AMIC^®^ after one year.	[98]
Autologous chondrocyte implantation (ACI)	113/97	8.4 ± 5.5	10 years	The Cincinnati score increased from 3.9 ± 1.5 to 6.4 ± 1.5, WOMAC improved from 39 ± 21 to 23 ± 16, KSS increased from 54 ± 18 to 79 ± 19, and KSS function increased from 65 ± 23 to 78 ± 17. The Physical Component increased from 33 ± 14 to 49 ± 18, and the Mental Component improved from 46 ± 14 to 52 ± 15.	[43]
Autologous minced cartilage (ACI)	15/12	3.1 ± 1.6 (1–6)	28.2 months	The decrease in pain (NAS) from 7.2 ±1.9 to 1.8 ± 1.6 and knee function improved (NAS) from 7.2 ± 2.0 to 2.1 ± 2.3. The preoperative AMADEUS score was 57.4 ± 21.4; postoperatively, MOCART was 40.6 ± 21.1.	[78]
Autologous minced cartilage (ACI) (chondrosphere)	53/22	4–10	3 years	The treatment was well tolerated. No differences in the incidence of any adverse events, or of patients with treatment-related adverse events, were observed.	[41]
Autologous chondrocyte implantation with spheroid	53/22	2–10	4 years	Overall KOOS scores showed a statistically significant improvement from 57.0 ± 15.2 to 77.1 ± 18.6.	[99]
ChonDux hydrogel scaffold	8/10	2 to 4	12, 24 months	ChonDux maintained durable tissue restoration over 24 months with a final defect percent fill of 94.2% ± 16.3% and no significant loss of fill volume at any time points. Tissues treated with ChonDux maintained T2 relaxation times similar to uninjured cartilage between 12 and 24 months. VAS pain scoring decreased between 1 and 6 weeks, and IKDC knee function scores improved by approximately 30.1 with ChonDux over 24 months.	[100]
osteochondral autologous transplantation (OAT)	14/7	0.15–2.8	4.4 years	All athletes were able to return to sport at their previous level; they were satisfied or very satisfied with their surgical outcome. The mean postoperative IKDC score was 84.5 ± 9.5. The mean Tegner score prior to injury was 8.9 ± 1.7; it was 7.7 ± 1.9 at final follow-up.	[101]
osteochondral autologous transplantation (OAT)	610	0.9–20.0	10.2	Overall, OAT showed successful outcomes in 72% of patients at long-term follow-up. Increased age, previous surgery, and defect size positively correlated with the failure rate, whereas success improved with concomitant surgical procedures.	[60]
Osteochondral allograft transplantation (OCA)	65/57	n.g.	13.5 years (2.4–10)	Improvement in pain and function, with graft survivorship of 82% at 10 years.	[65]
Osteochondral allograft transplantation (OCA)	30/25	n.g.	1.9–22.6 years	Pain and function improved on all outcome scales. 86% of patients were “extremely satisfied” or “satisfied”. OCA survivorship was 89.5% at 5 years and 74.7% at 10 years. At latest follow-up, OCA remained in situ in 82%.	[63]
Autologous osteochondral transplantation (OCA)	9/6	1.5–4.5	24 months	IKDC subjective score increased from 34.5 ± 23.6 to 66.3 ± 26.4. LKS increased from 47.8 ± 29.5 to 79.8 ± 24.6. The Tegner score increased at the 2-year follow-up, with stable results up to the last follow-up.	[102]
Arthroscopic mosaicplasty (OCA)	19/7	1.9 ± 0.6	12 years	IKDC subjective score increased from 36.8 ± 13.0 to 77.3 ±20.6; the Tegner score increased from 2.9 ± 1.3 to 5.2 ± SD 2.5. Better results were observed in patients with a higher pre-injury activity level and those requiring fewer plugs.	[103]
Autogenous osteochondral mosaicplasty (OCA)	16/6	0.9–3.0	9 years	84% of patients were satisfied or very satisfied with the procedure. The average IKDC was 74.5 ± 18.5 points. The average Lysholm score was 87.3 ± 11.6 points. The average Tegner score ranged from 6.35 ± 1.53 points prior to surgery to 5.60 ± 1.64 points after surgery. The MRI of 21 patients showed complete osteointegration of the grafts in 90% of cases.	[104]
Polydactyly-derived allogeneic chondrocyte cell-sheet transplantation	4/6	4–6	12 months	LKS improved from 40.1 ± 13.9 to 80.5 ± 15.7 points at the final follow-up. KOOS subscales (symptom, pain, function in daily living, function in sport and recreation, and quality of life) significantly improved.	[105]
Adipose tissue derived mesenchymal stem cell (AD-MSCs) LD: low-dose 1.0 × 10^7^ cells MD: mid-dose 5.0 × 10^7^ cells HD: high-dose 1.0 × 10^8^ cells	18 18 18	n.g.	6 months	No significant improvement of WOMAC and VAS for LG and MG. WOMAC decreased from 54.2 ± 5.2 to 32.8 ± 6.3; VAS decreased from 79.6 ± 2.2 to 44.2 ± 6.3 in HG. KSS increased in LD from 41.3 ± 6.8 to 79.0 ± 12.5, in HD from 47.2 ± 2.6 to 71.0 ± 4.4, and in LD from 60.0 ± 5.8 to 83.3 ± 8.8. The cartilage defect decreased in HG from 497.9 ± 29.7 mm^2^ to 297.9 ± 51.2 mm^2^ in the medial femoral condyle, from 333.2 ± 51.2 mm^2^ to 170.6 ± 48.2 mm^2^ in the medial tibial condyle, from 103.6 ± 27.1 mm^2^ to 51.1 ± 24.9 mm^2^ in the lateral femoral condyle, and from 19.4 ± 7.3 mm^2^ to 10.4 ± 4.2 mm^2^ in lateral tibial condyle. The cartilage volume increased in HG from 3.313 ± 0.304 cm^3^ to 3.78 ± 0.284 cm^3^ in the medial femoral condyle and from 1.157 ± 0.145 cm^3^ to 1.407 ± 0.150 cm^3^ in the medial tibial condyle.	[68]

AOFAS—The American Orthopaedic Foot and Ankle Society, VAS—the visual analogue scale, AAS—the visual analogue scale, LKS—Lysholm Knee Score, MRI—magnetic resonance imaging, KSS—Knee Society Clinical Rating System score, WOMAC—Western Ontario and McMaster Universities Osteoarthritis Index, AMADEUS—Area Measurement and Depth and Underlying Structures, MOCART—magnetic resonance observation of cartilage repair tissue, NAS—scores numeric analog scale, IKDC—International Knee Documentation Committee, ICRS II—the International Cartilage Repair Society Visual Assessment Scale II, KOOS—Knee injury and Osteoarthritis Outcome Score, n.g.—not given.

**Table 3:** Comparison of clinical outcomes for different surgical approaches in the treatment of knee cartilage defects.

Treatment	Patients (m/f)	Lesion Size (cm^2^)	Follow-Up	Clinical Outcome	Ref.
MIX microfracture mosaicplasty	14/6 14/6	2 to 6	1–15 years	Microfracture: LS improved 11 points (from 50 to 61). Mosaicplasty: LS improved 21 points (from 56 to 10,077). LS was higher in the mosaicplasty group than the microfracture group.	[59]
Microfracture (MFx), ACI-P (first-generation periosteum covered ACI)	12/10 12/10	2.37 ± 1.6 4.74 ± 2.3	10 years	NAS pain decreased from 7.2 ± 2.3 to 2.4 ± 2.4 and NAS function increased from 3.6 ± 2.7 to 8.1 ± 3.5 in MFX, and did not differ in ACI-P. LS increased from 43 ± 22 to 82 ± 15 (MFX) and from 42 ± 25 to 71 ± 18 (ACI-P). Lysholm and functional NAS scores were higher in MFX. IKDC, KOOS, and MOCART scores did not show differences between MFX and ACI-P.	[106]
ACI using spheroids, microfracture	33/19 28/22	2.7 ± 0.8 2.4 ± 0.8	5 years	The overall KOOS and its five subscores, MOCART, modified Lysholm, and IKDC were improved in both groups. In the ACI group, more rapid initial improvement of the KOOS was found compared to the microfracture group.	[47]
ACI using spheroids, microfracture	4/1	n.g.	16 months	Excellent histological results regarding the regeneration of hyaline articular cartilage were observed.	[49]
ACI using spheroids, microfracture	120	n.g.	1 year	The general response rate following spheroid-based ACI treatment was 75%, while the subset of patients treated with chondrocyte spheroids produced according to the stricter cultivation times exhibited a responder rate of 87%.	[50]
ACI using spheroids, microfracture	53/22	4–10	5 years	The overall KOOS showed significant improvement, increasing from a baseline score of 57.0 ± 15.2 to 73.4 ± 17.3 at the 1-year follow-up (*p* < 0.0001) and further to 76.9 ± 19.3 at the 5-year follow-up (*p* < 0.0001), regardless of the administered dose.	[51]
procedure IMPACT (Instant MSC Product Accompanying Autologous Chondron Transplantation)	24/11	3.2 ± 0.7	5 years	The majority of patients exhibited both statistically significant and clinically substantial enhancements in KOOS and its individual subscales from baseline to the 60-month mark: overall, scores increased from 57.9 ± 16.3 to 78.9 ± 17.7; Pain, from 62.3 ± 18.9 to 79.9 ± 20.0; Function, from 61.6 ± 16.5 to 79.4 ± 17.3; Activities of Daily Living, from 69.0 ± 19.0 to 89.9 ± 14.9; Sports and Recreation, from 32.3 ± 22.6 to 57.5 ± 30.0; and Quality of Life, from 25.9 ± 12.9 to 55.8 ± 26.8.	[56]
Matrix-Assisted Chondrocyte Implantation (MACI) microfracture (MFx)	45/27 48/24	>3	2-year	KOOS pain and function were better (*p* = 0.001) for MACI. KOOS activities of daily living (*p* < 0.001), quality of life (*p* = 0.029), and other symptoms (*p* < 0.001) were better for MACI. Repair tissue quality was good (histology/MRI) for MACI vs. MFx and there were no differences between the groups.	[107]
matrix-associated chondrocyte implantation (MACI) microfracture (MFx)	33/19 28/22	2.2 ± 0.7 2.0 ± 0.8	5 years	The overall KOOS and its five subscores, MOCART, modified Lysholm, and IKDC were improved in both groups. Non-inferiority of MACI to MFx was confirmed for the overall KOOS and the subscores, while the subscores for the activities of daily living, quality of life, and sports and recreation were better for MACI. In the MACI group, more rapid initial improvement of KOOS was found at three months for the older age group compared to the younger age group and MFx.	[47]
Autologous mesenchymal stromal cell (MSC) hialuronic acid (HA)	6/9 5/10	n.g.	12 months	The MSC group better improvement in algofunctional indices vs. HA. Significant decrease in poor cartilage areas, with cartilage quality improvements in MSC (T2 relaxation tests).	[69]
microfracture (MFx) osteochondral autologous transplantation (OAT)	6/5 8/6	2.0–5.2 2.0–6.0	9.8 years	There were no significant differences in the Lysholm score, KOOS, isokinetic muscle strength, or radiographic osteoarthritis between MFx and OAT. The mean Lysholm score at follow-up was 69.7 for the MFx group and 62.6 for OAT.	[108]
Microfracture (MFx) MFx with collagen	0/14 1/13	2.4–4.3	12 months	The quality of cartilage repair MFx with collagen was better than for MFx alone (ICRS II 1053.2 vs. 885.4; MOCART 64.6 vs. 45.4). VAS for pain, KOOS, IKDC, and Tegner activity scale scores were improved in both groups without differences between the groups.	[109]
Aragonite-Based Scaffold (ABS) Microfracture (MFx)	107/60 51/33	1–7	6, 12, 18, and 24 months	ABS showed a statistically superior outcome: the magnitude of improvement in ABS was twice as large as that in MFx in terms of mean KOOS improvement at 2 years. Overall, KOOS was 77.8% ABS vs. 33.6% in MFx. Statistically superior results were seen in the IKDC score as well. At 24 months, 88.5% of ABS had at least a 75% defect fill on magnetic resonance imaging as compared with 30.9% of MFx.	[110]
BST-CarGel^®^ a chitosan scaffold Microfracture (MFx)	22/12 14/12	2.41 ± 1.5 2.08 ± 1.2	5 years	The BST-CarGel^®^ group showed a better treatment effect for lesion filling and for repair tissue T2 relaxation times vs. MFx. BST-CarGel^®^ and MFx showed highly significant improvement in WOMAC and there were no differences between the groups. Safety was comparable for both groups.	[36]
Polydactyly-derived allogeneic chondrocyte cell-sheet transplantation	4/6	4–6	12 months	LKS improved from 40.1 ± 13.9 to 80.5 ± 15.7 points at the final follow-up. KOOS subscales (symptom, pain, function in daily living, function in sport and recreation, and quality of life) significantly improved.	[105]
Microfracture (MFx) + platelet-rich plasma alone or combination with adipose-derived mesenchymal stromal cells (AD-MSCs)	12/7 9/10	<2	1, 3, 6, and 12 months	No statistically significance in clinical outcomes (IKDC, KOOS, SF12, VAS) between the groups. The regenerated cartilage showed more hyaline-like features (higher collagen content, increased mineralization degree, and a higher number of viable cells) in the PRP + MSCs group.	[111]
costal chondrocyte–derived pellet-type autologous chondrocyte implantation (CCP-ACI) microfracture (MFx)	14/6 3/7	3.5 ± 1.4 2.5 ± 0.4	8, 24, and 48 weeks	MOCART scores improved and the improvement in the CCP-ACI was greater than that in the MFx group at 24 and 48 weeks (39.1 vs. 21.8 and 43.0 vs. 24.8, respectively). The proportions of complete defect repair and integration were higher for the CCP-ACI improvement in LKS and KOOS and knee-related quality of life was greater in the CCP-ACI than the MFx group (35.4 vs. 31.5, 35.7 vs. 28.5, and 27.9 vs. 11.6, respectively).	[112]
Matrix-Associated Autologous Chondrocyte Implantation (MACI) microfracture (MFx)	33/19 28/22	2.2 ± 0.7 2.0 ± 0.8	3 years	Significant improvement for both groups For the overall KOOS and for the subscores: Activities of Daily Living and Sport and Recreation and the superiority of MACI over MFx were shown at the descriptive level.	[113]
microfracture (MFx) Augmentation Using Cartilage Allograft Extracellular Matrix	37/11	≥1	2 years	All joint-specific and function-related patient-reported outcomes (PROs) significantly improved, apart from Marx activity, which declined in postoperative scores at 2 years. The percentage of patients achieving CSOs at 2 years was 90% for minimal, clinically important difference and 85% for patient acceptable symptomatic state. Two-year postoperative MRI demonstrated a mean 40.5 ± 22.9 MOCART score.	[114]
umbilical cord blood–derived mesenchymal stromal cells and 4% hyaluronate (UCB-MSC-HA) microfracture (MFx)	15/28 16/30	2–6	2, 3 and 5 years	Improvement by 1 ICRS grade was seen in 97.7% of UCB-MSC-HA vs. 71.7% of MFx. The overall histologic assessment score was better in the UCB-MSC-HA group. Improvement in VAS pain, WOMAC, and IKDC scores were not significantly different between the groups; however, the clinical results were significantly better in the UCB-MSC-HA group at 3- to 5-year follow-up. There were no differences between the groups in adverse events	[115]
microfracture (MFx) porcine-derived collagen-augmented chondrogenesis technique (C-ACT)	9/35 12/33	4.67 ± 2.54 3.98 ± 1.94	12, 24 months	The MOCART scores in C-ACT showed improved defect repair and filling, integration with the border zone, and effusion. MRI outcomes showed that the odds ratio (OR) for ≥50% defect filling at 12 months was statistically higher in the C-ACT. The likelihood of the RT/RC OR becoming ≥1 was significantly higher in the C-ACT. Postoperatively, at 24 months, the OR for the VAS 20% improvement rate was higher in C-ACT.	[116]

AOFAS—The American Orthopaedic Foot and Ankle Society, VAS—the visual analogue scale, AAS—the visual analogue scale, LKS—Lysholm Knee Score, MRI—magnetic resonance imaging, KSS—Knee Society Clinical Rating System score, WOMAC—Western Ontario and McMaster Universities Osteoarthritis Index, AMADEUS—Area Measurement and Depth and Underlying Structures, MOCART—magnetic resonance observation of cartilage repair tissue, NAS—scores numeric analog scale, IKDC—International Knee Documentation Committee, ICRS II—the International Cartilage Repair Society Visual Assessment Scale II, KOOS—Knee Injury and Osteoarthritis Outcome Score, n.g.—not given.

## Data Availability

Data are contained within the article.
